# The Differential Role of Executive Apathy in Alzheimer’s Disease Dementia, Mild Cognitive Impairment and Healthy Cognitive Ageing

**DOI:** 10.3390/geriatrics8020038

**Published:** 2023-03-16

**Authors:** Michalis Mougias, Ion N. Beratis, Kleio Moustaka, Panagiotis Alexopoulos, Konstantinos Assimakopoulos

**Affiliations:** 1 Alzheimer’s Center, “Nestor” Greek Psychogeriatric Association, 22, Ioanni Drossopoulou Street, 112 57 Athens, Greece; drmougias@gmail.com (M.M.); k.moustaka.ps@gmail.com (K.M.); 2 Department of Psychiatry, School of Medicine, University of Patras, 265 04 Patras, Greece; panos.alexopoulos@upatras.gr (P.A.); kassima@upatras.gr (K.A.); 3 Psychology Department, The American College of Greece, Deree, 6, Gravias Street, 153 42 Athens, Greece; 4 Department of Psychiatry and Psychotherapy, Klinikum Rechts der Isar, Technical University of Munich, 21, Arcisstraße Street, 80 333 Munich, Germany

**Keywords:** emotional apathy, executive apathy, initiation apathy, Alzheimer’s disease, mild cognitive impairment, dimensional apathy scale

## Abstract

The objective of the present work was to compare the levels of executive, emotional, and initiation apathy in individuals with mild cognitive impairment (MCI), mild Alzheimer’s disease dementia (ADD), and cognitively intact healthy controls (HCs). Fifty-two patients with mild ADD, 40 individuals with MCI, and 37 cognitively intact individuals were included in the current study. The participants were consecutive visitors to the Outpatient Memory Clinic of “Nestor” Alzheimer’s Center. The symptoms of apathy were measured with the dimensional apathy scale. Analyses showed that ADD patients had significantly higher degrees of executive, emotional, initiation, and overall apathy compared with both the MCI group and the HCs. Additionally, a significant difference was observed in the dimension of executive apathy between individuals with MCI and the HCs. In conclusion, the dimension of executive apathy was the most sensitive measure regarding the differentiation of individuals with mild ADD or MCI and HCs. Hence, detailed evaluation of executive apathy in older individuals referred to a memory clinic may provide useful information contributing to their diagnostic categorization and to the differentiation between neurocognitive disorders and healthy cognitive ageing.

## 1. Introduction

Neurodegenerative diseases have been in the spotlight of contemporary medicine, due to the increase in life expectancy observed in recent decades, especially in the Western world. Dementia is defined as a progressive cognitive disorder, which commonly affects older people. Along with cognitive decline, it also involves significant impairment in functionality and interpersonal relationships [1]. Except for frontotemporal dementia, most forms of dementia occur in people over 60 years old, and often present initially with memory impairment that may affect the ability to learn new information. Alzheimer’s disease (AD) is the most common cause of dementia. It is estimated that 5–10% of older people (>65 years) suffer from the Alzheimer’s disease dementia (ADD), and this percentage increases to 20–40% for those aged >85 years [2]. In Greece, 9% of people over the age of 70 suffer from dementia, while 6% suffer from ADD [3].

One of the clinical features of AD is its insidious onset, which is followed by the progressive deterioration of cognition and functionality. Because the progression of AD is gradual, both clinical practice and epidemiological research commonly focus on patients in the pre-dementia phase who manifest attenuated memory ability and/or other cognitive functions above the expected level, but in whom the criteria for the diagnosis of dementia are not yet met [4]. As ADD results from neurodegenerative processes that begin well before the symptoms’ onset, and since its treatment is not sufficiently effective after the disorder is established, scientific research has focused on the transition from normal aging to dementia. In this context, the concept of mild cognitive impairment (MCI) has been introduced, referring to the intermediate stage between normal cognition and dementia [5,6,7]. MCI has been divided into two major subcategories: the amnestic type of MCI (aMCI) and the non-amnestic type (naMCI), according to its symptomatology. According to Petersen et al. (2009), aMCI is presented with memory impairment of clinical significance, while naMCI manifests as clinically significant deterioration of one or more memory-unrelated domains of cognition [8]. Moreover, MCI may also be differentiated into single-domain MCI (sdMCI; one cognitive domain affected) and multiple-domain MCI (mdMCI; more than one cognitive domain affected), based on the number of cognitive domains affected during the course of the disease [8].

At the clinical stages of dementia and MCI, the cognitive symptomatology observed may be accompanied by various behavioral and psychological symptoms of dementia (BPSDs), including irritability, anxiety, depression, delusions, hallucinations, and aggression, with apathy being the most common BPSD [9,10,11,12]. Studies have shown that BPSDs may occur at any stage of the disease, and that approximately 96% of patients with ADD had at least one of these symptoms at the time of initial evaluation [13,14].

Studies have investigated BPSDs in AD and MCI [15]. Depressive symptoms were present in patients with AD and MCI, while apathy was more common in AD compared with MCI. Age and degree of depression have been associated with apathy in ADD patients [16]. This link was preserved even after controlling for relevant factors, such as age, degree of function, and baseline cognition [17]. An indicative prospective study [18] investigated the association between emotional symptoms and the probability of AD in patients with MCI. Depressive symptoms in individuals with MCI were associated with increased risk of transition to ADD. Similarly, individuals with MCI that have increased levels of apathy, with rates ranging from 11.1% to 39.8%, appear to be at accentuated risk of progression to ADD [19,20,21,22,23,24,25,26]. Recent findings also reveal that MCI patients experiencing apathy alone, or both depression and apathy, have a higher risk of progression to AD than patients without BPSDs [27,28,29]. In addition, previous research indicates that patients with multiple-domain aMCI more commonly show symptoms of apathy compared to patients with single-domain aMCI [14]. On the other hand, the presence of greater cognitive reserve, as reflected by relevant indicators such as high educational attainment, language skills, and frequent cognitive or social activities in patients with MCI, appears to be a protective factor against the development of dementia [30,31]. Furthermore, it appears that positive cognitive reserve indicators enhance the possibility of reversion to normal cognitive functioning in patients with MCI [32].

Apathy is considered a form of executive cognitive dysfunction [33], which includes psychological as well as behavioral aspects [34]. In this regard, Marin (1991) defines apathy as lack of motivation, diminished goal-directed cognition, behavior, and mobilization [35]. People with apathy “do less, think less and feel less”. Moreover, apathy may appear as an element of the clinical sequelae of various mental disorders beyond dementia and MCI, such as schizophrenia [36,37,38,39,40]. The spectrum of apathy-related symptoms includes decreased levels of initiative, interest, motivation, mobilization, spontaneity, energy, enthusiasm, expression of emotions, and perseverance [41,42].

Stuss et al. (2000) suggest the existence of three subtypes of apathy: emotional, cognitive, and behavioral, each of which is determined by different anatomical features and psychological mechanisms [43]. Radakovic and Abrahams (2014) developed the dimensional apathy scale (DAS), which assesses the following three subtypes of apathy: executive (ExA), emotional (EmA), and behavioral/cognitive initiation (InA) [44]. This taxonomy was based on a thorough review of relevant research findings [45,46]. ExA refers to the inability to manage goals, inadequate action planning, and attenuated skills relating to strategy and organization. EmA reflects diminished integration, processing, and expression of emotional behaviors, resulting in a continuous lack of vivid and spontaneous affect. InA indicates decreased initiation of thoughts or behaviors, with a direct impact on various elements of functionality. At a neuroanatomical level, certain studies have explored the association between apathy and the integrity of specific brain regions. Raimo et al. (2019) connected apathy with the presence of dysfunction in cortical areas related to regulation of emotion and executive processing [47]. Moreover, Gonçalves et al. (2020) showed that severity of apathy was negatively associated with grey matter volume in the medial prefrontal cortex—an area involved in rewards-related effort-rooted behavior [48]. Furthermore, apathy has been neuroanatomically correlated with loss of grey matter in frontal, limbic, and temporal areas [49].

The most common clinical causes of apathy are various neurodegenerative disorders [50]. Apathy is a major challenge in dementia, as it is one of the most frequent and persistent behavioral symptoms [33], especially of the subcortical types of dementia [51,52,53,54,55]. In studies using the Neuropsychiatric Inventory (NPI), the rate of apathy reported in patients with ADD reaches up to 70% [56,57]. The latter indicated that apathy is present in 28% of patients with mild AD, and this rate rises drastically in patients with severe ADD. 

The current literature is limited in its multidimensional examination of apathy, and is mainly focused on patients with amyotrophic lateral sclerosis (ALS), Huntington’s disease, or Parkinson’s disease [58,59,60]. Although studies have examined the multidimensional model of apathy as it uniquely appears in ADD, in the behavioral variant of frontotemporal dementia (bvFTD), and in primary progressive aphasia (PPA) [60,61,62], to the best of our knowledge, no previous studies have focused on assessing the various dimensions of apathy in patients with MCI. Evidence reveals a greater effect size in terms of differences in ExA than in EmA and InA, in ADD patients compared with controls [62,63]. 

The present study aimed to examine the different dimensions of apathy in the following three groups: mild ADD patients, MCI patients, and HCs. According to previous findings, it was hypothesized that patients with ADD will have significantly greater levels of apathy, in terms of both the overall index (global apathy) and of the three subtypes, namely executive, emotional, initiation [60]. Our capacity to formulate a strong directional hypothesis was restricted by the lack of previous research comparing patients with MCI and cognitively intact individuals. Nonetheless, based on previous findings that indicated a greater effect size on ExA when comparing patients with ADD and HCs [62,63], it was expected that ExA would also be the most sensitive index for individuals with MCI. Furthermore, since previous research indicates that patients with multiple-domain aMCI exhibit elevated symptoms of apathy [14], we also expected to observe a similar pattern of results in this study. Additionally, the current work explored the level of shared variance between the classical measure of apathy (NPI) and the components of the multidimensional model (DAS). Finally, the relationship was explored between the three dimensions of apathy and negative mood (depression, anxiety, stress), to increase our insight regarding the BPSDs in MCI and mild ADD patients.

## 2. Methods

### 2.1. Participants

Overall, the sample of the present quasi-experimental study comprised 129 individuals (Age: mean = 74.74, SD = 6.89; Education: mean = 10.61, SD = 4.87; 85 females), categorized into three distinct groups. Specifically, the first group included 52 patients diagnosed with ADD (Age: mean = 77.48, SD = 7.17). The second group included 40 MCI patients (mean = 73.55, SD = 5.31), and the third group included 37 HC individuals (HCs; Age: mean = 72.16, SD = 6.80). According to the G-Power software, the power of the study for detecting medium effect sizes was found to be at the level of 0.86 for the specific sample size. The healthy control group consisted of individuals who visited the clinic only once and for the purpose of prevention, without having any memory complaints. They were the caregivers of or close friends with people who suffer from dementia, and were not genetically related to them. The individuals in this group were considered cognitively intact, since their scores for a set of cognitive tests were within the normal range based on local norms that take into consideration age and level of education. Petersen and Morris’ [64] criteria were applied for the MCI diagnosis, including complaints of memory impairment from patients or a family member, verified impairment in at least one cognitive domain, but with preserved functional abilities of daily living and absence of dementia. According to the neuropsychological evaluation, all participants with MCI met the criteria for the amnestic subtype. Fifteen participants met the criteria for the single-domain amnestic subtype and 25 participants met the criteria for the multiple-domain amnestic subtype of MCI. The AD diagnoses were made following McKhann’s criteria [65]. Furthermore, the inclusion criteria for participation in this study were: MMSE score of at least 20, Greek native language, absence of psychiatric illness or chronic and incurable organic disease, diagnosis of AD for the dementia group, and absence of accommodation in a nursing home or hospitalization for at least one week before the evaluation. Since our goal was to detect patterns of change in apathy levels at the early stages of dementia, the aforementioned criteria regarding the MMSE score and the absence of hospitalization were applied with the aim of excluding patients with more advanced stages of ADD.

### 2.2. Materials

The diagnostic workup of the study sample included the following well-established and relevant instruments.

*Mini Mental State Examination (MMSE).* The MMSE is a test of general cognitive status [66,67]. The components that are assessed include: (a) attention, (b) time and space orientation, (c) memory, (d) language, and (e) visuospatial skills. Scores may range from 0 (high cognitive impairment) to 30 (no cognitive impairment). According to a large sample of 1114 participants, it appears that the mean value of the MMSE score for patients with MCI is typically close to 26 and the mean value of the expected MMSE score for patients with mild ADD is close to 22 [68].

*Katz Activities of Daily Living (Katz ADL).* This scale measures patients’ levels of autonomy in daily functioning [69]. An index of performance adequacy is calculated for the following six daily functions: bathing, dressing, feeding, continence, transferring, and toileting, with scores ranging from 0 (fully dependent) to 6 (fully independent).

*Instrumental Activities of Daily Living (Lawton IADL-8).* The Lawton 8-item IADL [70,71,72] evaluates patients’ daily functioning in the following eight areas: telephone use, daily shopping, meal preparation, home economics, washing clothes, transportation use, responsibility for taking medication, and money management. Scores range from 0 (fully independent) to 8 (fully dependent).

*Neuropsychiatric Inventory (NPI-Q).* The NPI-Q scale [73,74] examines the following 10 types of behavioral disturbances: delirium, delusions, aggression, depression, anxiety, euphoric mood, apathy, disinhibition, irritability, emotion variability, and abnormal motor behavior. Disturbances in sleep and eating may also be detected through this scale. Particularly, the scale measures frequency from 1 (never) to 4 (usually), weight from 1 (mild) to 3 (high), and dysphoria caused to the caregiver from 0 (none) to 5 (extreme), for each behavioral disturbance. 

*Depression Anxiety Stress Scale (DASS-21).* The three-dimensional brief DASS-21 scale is a questionnaire that measures the degree of psychological dysphoria regarding three axes: depression, anxiety, and stress [75,76]. In particular, the dimension of depression evaluates dysphoria, despair, life depreciation, self-depreciation, lack of interest/participation, and anhedonia. The anxiety dimension assesses arousal of the autonomous nervous system, its musculoskeletal effect, state anxiety, and subjective experience of anxiety. The stress axis evaluates restlessness, hyperarousal, temperament, irritability/hyperreactivity, and impatience. Each dimension has an overall score resulting from seven items, each of which is measured from 0 (none) to 3 (very much).

*Dimensional Apathy Scale (DAS).* The DAS assesses the three dimensions of apathy—emotional, executive, and initiation—based on both the patient and their caregiver [41,77]. It contains 24 items, each of which can be rated from 1 (almost never) to 4 (almost always). An overall score of apathy may vary from 0 to 72 (revealing a high degree of total apathy) and is calculated through summing up all individual scores of the 24 items. 

*Verbal Fluency Test (VFT).* The VFT evaluates verbal ability, as well as executive functions such as working memory, attention shifting, and inhibition [78,79]. Verbal fluency can be further categorized into semantic fluency, which is the retrieval ability of words from one category (animals, fruits, objects), and phonemic fluency, that is the retrieval ability of words starting from a specific letter (X, S, A). Patients are instructed to retrieve as many words as possible in one minute for each of the three sub-categories of each type of fluency.

*Rey Auditory Verbal Learning Test (RAVLT).* The RAVLT assesses memory and its three basic functions—coding, consolidation, and retrieval—in three phases [80,81]. The first phase contains 5 trials of learning 15 nouns (list A), and evaluates immediate recall, extracting a learning curve from the number of words recalled by the patient in each of the 5 trials. During the second phase (interference), a different list (B) of 15 nouns is presented, and immediate recall is again measured in a single trial. The next phase, taking place after approximately 30 min, measures patient’s delayed recall of list A in a single trial. Finally, the patient is asked to identify recognize the words in list A from a longer list. 

*Trail Making Test (TMT).* The TMT has two subtests, parts A (TMT-A) and B (TMT-B) [82]. Each subtest is presented on a white paper (A4 dimensions) and the participants are asked to connect circles in a certain order as fast as possible. Part A includes circles only with numbers (1–25) that must be connected in numerical order, while part B includes circles with numbers (1–13) and letters (A-Μ) that must be connected in ascending order by alternating between numbers and letters. Abilities such as visual search, motor speed, and spatial skills are assessed in both parts of the test. In addition, part B is responsible for assessing aspects of executive control, such as mental flexibility and task shifting [83].

### 2.3. Procedure

The study participants were consecutive visitors of the Outpatient Memory Clinic of Nestor Alzheimer’s Center, meeting the previously mentioned inclusion criteria. All individuals participated voluntarily and provided written consent. The overall data-collection process lasted approximately two hours, including a medical and a neuropsychological assessment. In particular, the medical assessment conducted by a psychiatrist/neurologist included a detailed medical history, a comprehensive screening for any neurological signs, and evaluation of the neuropsychiatric profile as well as of the level of functioning of each participant. The neuropsychological evaluation, conducted by neuropsychologists, assessed various cognitive domains such as general cognitive status, episodic memory, information processing speed, verbal fluency, executive functioning, as well as levels of insomnia and neuropsychiatric symptomatology.

### 2.4. Ethical Considerations

The study was approved by the Ethics Committee of the Alzheimer’s Center, “Nestor” Greek Psychogeriatric Association. Informed consent was obtained from all individuals that took part; it was explained to them that participation was on a voluntary basis and that they had the right to withdraw at any time. Participants were informed about the nature of the study, the duration of their engagement, and the type of information that they would be asked to provide during the data collection process. Furthermore, participants were notified about the confidentiality of the procedure and that the use of their background information would be only for research purposes. Participation was voluntary, and no compensation was offered.

### 2.5. Statistical Analyses

Descriptive statistics: Regarding the measures of descriptive statistics that were applied, the mean value and the SD were computed for the continuous variables of the study. For the categorical variables of the study, the absolute and relative frequency were utilized.

*Analyses of Variance and Covariance (ANOVA and ANCOVA).* ANOVA analyses were conducted to measure the impact of cognitive impairment (ADD, MCI, HC) on the overall DAS degree of apathy (OvA), as well as on its dimensions (ExA, EmA, InA). ANCOVA analyses were also utilized to investigate the aforementioned effects after controlling for the role of the DASS depression subscale score. For the models that were statistically significant, post-hoc comparisons were applied using the Bonferroni correction, in order to clarify which clinical groups in the study differed from each other significantly.

*Correlation Analyses.* Pearson correlation analyses were utilized to investigate relationships between scores for the DASS depression and anxiety subscales and the total degree of apathy, including its subscales (ExA, EmA, InA). Moreover, the same type of analysis was used to examine possible correlations between apathy as evaluated via the NPI scale and the DAS scale, including its three dimensions in the sub-group of patients with AD.

## 3. Results

### 3.1. Descriptive Statistics

The main characteristics (age, gender, education) of the overall sample and the three clinical groups are presented in Table 1 and Table 2, respectively. The descriptive statistics of the cognitive measures (MMSE, REY AVLT, verbal fluency, and trail making test scores) for each clinical group can be found in Table 3.

### 3.2. Degree of Apathy in Groups of Patients with ADD, MCI, and HCs

One-way ANOVA analyses revealed statistically significant effects of the clinical group (ADD, MCI, HC; see Table 4) on both OvA (F (2,126) = 22.46, *p* < 0.001), and its three sub-dimensions, i.e., ExA (F (2,126) = 17.21, *p* < 0.001), EmA (F (2,126) = 12.83, *p* < 0.001), and InA (F (2,126) = 16.40, *p* < 0.001).

Furthermore, one-way ANOVA analyses showed statistically significant differences between the three clinical groups (ADD, MCI, HC) in relation to the factors of depression (F (2,126) = 3.43, *p* = 0.025) and age (F (2,126) = 10.56, *p* < 0.001). Specifically, post-hoc statistical comparisons using the Bonferroni correction revealed that ADD patients exhibited significantly greater levels of depression compared with HC, *p* = 0.030, while no other differences were found in terms of DASS depression. Moreover, there were statistically significant differences in age between ADD patients and HCs, *p* < 0.001, as well as between ADD and MCI patients, *p* = 0.001. The group of ADD patients was older compared with MCI patients and HCs, while the last two clinical groups did not significantly differ from each other in terms of age.

### 3.3. Total Apathy

Post-hoc comparisons using the Bonferroni correction showed that ADD patients (mean = 35.60, SD = 10.71) had a significantly greater degree of OvA compared with MCI patients (mean = 24.00, SD = 10.95; *p* < 0.001) and HCs (mean = 21.69, SD = 7.26; *p* < 0.001). The pattern of differences in OvA between the three groups was maintained at statistically significant levels, even after controlling for depression and age as covariates, using an ANCOVA analysis, F (2,124) = 14.29, *p* < 0.001.

### 3.4. Executive Apathy

Post-hoc comparisons using the Bonferroni correction showed that ADD patients (mean = 11.88, SD = 4.93) had a significantly greater degree of ExA compared withMCI patients (mean = 8.84, SD = 4.41; *p* = 0.006) and HCs (mean = 5.86, SD = 3.34; *p* < 0.001). Moreover, results revealed that MCI patients had significantly greater levels of ExA compared with HCs (mean = 5.86, SD = 3.34; *p* = 0.021). The pattern of differences in ExA between the three groups was maintained at statistically significant levels, even after controlling for the role of depression and age as covariates, through running an ANCOVA analysis, F (2,124) = 11.15, *p* < 0.001.

### 3.5. Emotional Apathy

Post-hoc comparisons using the Bonferroni correction revealed significantly higher EmA for ADD patients (mean = 10.92, SD = 3.80) compared with MCI patients (mean = 7.34, SD = 3.58; *p* < 0.001) and HCs (mean = 7.79, SD = 3.03; *p* = 0.001). The pattern of differences in EmA was maintained at statistically significant levels among the three groups, even after controlling for the role of depression and age as covariates, using the ANCOVA test, F (2,124) = 6.22, *p* = 0.003.

### 3.6. Initiation Apathy

Post-hoc comparisons with Bonferroni correction revealed a significantly greater degree of InA for ADD patients (mean = 12.81, SD = 4.65) compared with MCI patients (mean = 7.82, SD = 5.03; *p* < 0.001), and HCs (mean = 8.03, SD = 3.51; *p* < 0.001). The pattern of differences in InA was maintained at statistically significant levels among the three groups, even after controlling for the role of depression and age as covariates, using an ANCOVA analysis, F (2,124) = 12.12, *p* < 0.001).

### 3.7. Comparison of Apathy Levels between Single-Domain Amnestic and Multiple-Domain Amnestic MCI

Independent samples *t*-test analyses were applied to compare the different dimensions of apathy between single-domain amnestic and multiple-domain amnestic MCI patients. The analyses revealed significantly greater levels of ExA (t = 2.25, *p* = 0.030) and OvA (t = 2.35, *p* = 0.024) in the group of multiple-domain amnestic MCI patients (see Table 5). For EmA and InA, the comparisons did not reach the level of statistical significance.

### 3.8. Relationship between Negative Affect and Apathy

Because the assumption of normality was not met according to Kolmogorov–Smirnov analysis, Spearman correlation analysis was applied to investigate the relationships between the DASS anxiety and depression subscale scores and OvA, including its subscales (ExA, EmA, InA; see Table 6, Table 7 and Table 8). Results revealed statistically significant correlations between depression and ExA (*p* < 0.001, r = 0.448), EmA (*p* = 0.002, r = 0.264), InA (*p* < 0.001, r = 0.322), as well as OvA (*p* < 0.001, r = 0.438). Particularly in the ADD group, depression was significantly correlated with both ExA (*p* < 0.001, r = 0.653) and OvA (*p* = 0.001, r = 0.486; see Table 6). In the MCI group, depression was significantly correlated with ExA (*p* < 0.027, r = 0.346).

Regarding anxiety, there was a statistically significant correlation with OvA (*p* = 0.006, r = 0.239), as well as with ExA (*p* < 0.001, r = 0.401; see Table 5). Specifically, in the ADD group, anxiety was significantly correlated with both ExA (*p* = 0.001, r = 0.458), and OvA (*p* = 0.031, r = 0.296; Table 8). 

### 3.9. Investigation of Different Measures of Apathy

Because the assumption of normality was not met according to Kolmogorov–Smirnov analysis, Spearman correlation analyses were carried out to examine the relationship between apathy as evaluated in the Neuropsychiatric Inventory (NPI) and apathy as calculated in the multidimensional scale (DAS) for ADD patients (the sample size for this analysis was 41, because 11 out of the 52 patients with ADD did not complete the NPI scale). Results revealed statistically significant correlations between NPI apathy and DAS ExA (*p* = 0.005, r = 0.428) and DAS InA (*p* < 0.001, r = 0.559). Furthermore, analysis revealed a statistically significant relationship between NPI apathy and DAS OvA (*p* = 0.001, r = 0.476). However, there was a marginally non-significant correlation between NPI apathy and DAS EmA (*p* = 0.051, r = 0.303). The coefficient of determination for each of the aforementioned correlations was as follows: NPI apathy with DAS ExA (R^2^ = 0.18), DAS EmA (R^2^ = 0.09), DAS InA (R^2^ = 0.31), and DAS OvA (R^2^ = 0.23). For more information, see Table 9.

## 4. Discussion

The main goal of the present study was to compare the levels of apathy between three groups: ADD patients, MCI patients, and HC individuals.

The results of the study highlight the importance of investigating the dimensions of apathy in patients with dementia. Specifically, as expected, ADD patients showed significantly higher degrees of ExA, EmA, InA, and OvA compared with both the MCI and the HC groups. Additionally, in line with the current study hypothesis, in the dimension of ExA a significant difference was observed between individuals with MCI and the HCs. Importantly, this pattern of differences between the groups was retained after controlling for the role of depressive symptoms and age. Meanwhile, those individuals within the MCI group that met the criteria for multiple-domain amnestic MCI showed higher levels of ExA and OvA compared with those that met the criteria for single-domain amnestic MCI. Finally, in the group of ADD patients, the maximum level of shared variance, as indicated by the value of the coefficient of determination, was approximately 30% between DAS InA and NPI apathy. However, the corresponding value was substantially lower in all other dimensions of DAS apathy; especially for EmA, where it was only 9%. 

The higher levels of apathy expressed in the ADD group are in line with previously accumulated findings supporting the presence of a positive association between apathy levels and the degree of the neurodegenerative process [60]. Nonetheless, to the best of our knowledge, the novel element of the current work is the multidimensional evaluation of apathy within the clinical spectrum of MCI. 

In this context, the comparison of the three dimensions of apathy between the clinical groups of the study revealed that the degree of ExA increased in a ‘dose-responsive’ pattern as the level of cognitive dysfunction increased, i.e., the ADD group showed higher levels than the MCI group, and similarly the latter showed higher levels than the HCs. This finding supports the hypothesis of the study, that expected a higher degree of ExA in the MCI group compared to HCs. The underlying substrate for developing the specific hypothesis was based on previous findings showing a greater effect size on ExA in comparison between patients with ADD and HCs [62,63]. Therefore, it was expected that ExA would serve as the most sensitive index in the comparison between the MCI group and the HCs. Moreover, the increased levels of ExA and OvA that were observed in cases of multiple-domain amnestic MCI, as compared with single-domain amnestic MCI, are in line with the findings of previous relevant research [14] and further indicate that elevated levels of the specific forms of apathy seem to be present in patients who are at higher risk of progression to dementia [84,85]. The other subtypes of apathy did not show this pattern, as the ADD group displayed higher levels for both EmA and InA. However, the MCI and HC groups manifested similar levels of apathy in these two dimensions. This pattern of findings supports the need for detailed evaluation of ExA symptoms in MCI patients, which appear to be more commonly accentuated in the specific clinical group compared with symptoms of EmA and InA. In addition, future longitudinal studies should explore whether the presence of elevated symptoms of ExA increases to a greater extent the risk of developing AD, compared with the presence of other forms of apathy.

The importance of differentiating between the subtypes of apathy is also reflected in their corresponding associations with depression and anxiety. Specifically, in patients with ADD the degree of anxiety and depression was positively associated with ExA and OvA. To the best of our knowledge, this was the first study to have explored the aforementioned associations by using the multidimensional model of apathy in this specific clinical group. Nonetheless, previous studies have significantly associated unified measures of apathy with depression and anxiety [86,87,88,89]. The present findings could be explained through the negative impact of ExA on action planning and motivation—a condition that could trigger or enhance symptoms of depression and anxiety in everyday life. Therefore, future studies could strengthen the current findings by further exploring the underlying pattern of associations between the various dimensions of apathy and other BPSDs. On the other hand, in the group of individuals with MCI the only significant association was between depression and ExA, but this was less strongly expressed compared with the ADD group. This observation is at least in moderate agreement with previous studies that support the distinct presentation of apathy and depression in the MCI population [90,91]. Possibly, this finding is also related to the lower levels of apathy found in MCI compared with ADD patients; a parameter that could at least partially explain the reduced shared variance between depression and apathy observed in the former clinical group. 

Another objective of the current study was to explore the strength of the associations between the classical NPI measure of apathy, and the various dimensions of apathy as assessed by the DAS scale in the ADD group. The findings revealed significant associations in all cases, except for EmA. The strongest association was observed between InA and NPI apathy, with the amount of shared variance between the two scales reaching a level of 31%, as indicated by the corresponding value of the coefficient of determination. This therefore implies that the application of more specialized measures of apathy could provide additional information regarding patients’ apathy-related symptomatology that cannot be captured by the classical NPI scale, especially in the case of ExA (18% shared variance with NPI) and EmA (9% shared variance with NPI).

A limitation of this work that should be noted is the relatively small sample that did not allow exploration of the role of various subject-related variables, including gender, education, age, and further evaluation of MCI type by including non-amnestic patients, in relation to the levels and patterns of apathy that were observed. This direction of research is important for strengthening the conclusions of the present study, because a trend was revealed for lower education in ADD and MCI patients compared with HC individuals, and there were a greater number of female participants in the obtained sample. Previous findings have shown that male individuals with MCI may have a greater likelihood of developing symptoms of apathy, compared with females [92], as well as a tendency for significant interaction between gender and age to affect their levels of apathy [93]. Therefore, future studies could increase these insights and strengthen the current findings by investigating the effect of these factors and their interactions on the different dimensions of apathy observed in individuals with ADD or MCI. In addition, a reasonable target for future research could be the identification of those forms of apathy that influence more drastically the risk for progression to dementia. Prospective studies could focus on exploring the capacity of the various forms of apathy to predict quality of life for individuals with MCI as well as their family members. Furthermore, because there are some signs of evidence suggesting a negative association between enhanced levels of cognitive reserve and apathy in patients with MCI [94], future studies should further explore the architectonics of this relationship by additionally focusing on the different components of apathy.

In conclusion, the findings of this study support the need for careful assessment of the various dimensions of apathy in individuals with a diagnosis of ADD or MCI, medical conditions with considerable prevalence above the age of 65. This is especially the case for the dimension ExA, which was the most sensitive measure for the differentiation of individuals with mild ADD, MCI and HCs. Furthermore, the association observed in the current study between the various subtypes of apathy and other neuropsychiatric symptoms could create a stimulating base for future research in this direction. Finally, these observations may have considerable practical importance as they provide useful information regarding the profile of apathy in cases of individuals with MCI, as well as on the development of timely individualized interventions that can enhance quality of life for these vulnerable members of our society. 

## Figures and Tables

**Table 1:** Sociodemographic characteristics of participants (N = 129).

Participants’ Characteristics	Number of Participants	Statistics
	N (%)	Mean (SD)
Overall Sample		
Age		74.74 (6.89)
Education		10.61 (4.87)
Gender		
Female	85 (65.90)	
Male	44 (34.10)	

**Table 2:** Sociodemographic characteristics of the clinical groups.

Characteristics	Clinical Groups					
	ADD	MCI			HC	
	N (%)	M (SD)	N (%)	M (SD)	N (%)	M (SD)
Age		77.48 (7.17)		73.55 (5.31)		72.16 (6.80)
Education		9.87 (4.64)		10.53 (4.99)		11.76 (4.96)
Gender						
Female	28 (53.80)		29 (72.50)		28 (75.70)	
Male	24 (46.20)		11 (27.50)		9 (24.30)	

Note: ADD: N = 52 (40.31%), MCI: N = 40 (30.01%), HC: N = 37 (28.68%).

**Table 3:** Cognitive measures of the clinical groups.

Characteristics	Clinical Groups		
	ADD	MCI	HC
	M (SD)	M (SD)	M (SD)
Verbal Fluency			
Semantic	25.71 (9.08)	38.13 (12.05)	49.29 (10.44)
Phonemic	18.02 (9.18)	28.53 (11.23)	32.62 (10.31)
MMSE	22.32 (3.76)	26.75 (2.05)	28.95 (1.05)
REY AVLT Test			
A5	6.52 (2.07)	8.43 (2.10)	12.12 (2.00)
Delayed Recall	1.71 (1.84)	4.23 (2.47)	9.56 (2.68)
Recognition	4.71 (2.91)	6.73 (4.10)	11.65 (2.29)
Trail Making Test			
Trail A	118.67 (50.35)	78.58 (34.49)	62.97 (17.74)
Trail B	268.90 (69.66)	183.05 (65.63)	125.03 (44.91)

Note: ADD: N = 52 (40.31%), MCI: N = 40 (30.01%), HC: N = 37 (28.68%).

**Table 4:** Apathy comparisons among the different clinical groups.

			HC	MCI	ADD
DAS Scale	Clinical Group	Mean (SD)	Sig.		
Total Apathy	HC	21.69 (7.26)	-	1.000	<0.001
	MCI	24.00 (10.95)	1.000	-	<0.001
	ADD	35.60 (10.71)	<0.001	<0.001	-
Executive Apathy	HC	5.86 (3.34)	-	0.021	<0.001
	MCI	8.84 (4.41)	0.021	-	0.006
	ADD	11.88 (4.93)	<0.001	0.006	-
Emotional Apathy	HC	7.79 (3.03)	-	1.000	0.001
	MCI	7.34 (3.58)	1.000	-	<0.001
	ADD	10.92 (3.80)	0.001	<0.001	-
Initiation Apathy	HC	8.03 (3.51)	-	1.000	<0.001
	MCI	7.82 (5.03)	1.000	-	<0.001
	ADD	12.81 (4.65)	<0.001	<0.001	-

Note: ADD (Alzheimer’s disease dementia), MCI (mild cognitive impairment), HC (cognitively intact healthy controls).

**Table 5:** Apathy measures of the different MCI types.

DAS Measures	MCI Types	
	Single-Domain Amnestic	Multiple-Domain Amnestic
	M (SD)	M (SD)
Overall Apathy	19.00 (9.20)	26.85 (10.95)
Executive Apathy	6.80 (3.82)	9.85 (4.40)
Emotional Apathy	6.00 (3.14)	8.04 (3.63)
Initiation Apathy	6.20 (4.74)	8.96 (5.27)

**Table 6:** Overall correlations between DAS apathy and DASS depression and anxiety.

DAS	Depression	Anxiety
Total Apathy	0.438 ***	0.239 ***
Executive Apathy	0.448 ***	0.401 ***
Emotional Apathy	0.264 ***	0.034
Initiation Apathy	0.322 ***	0.073

Note 1: *** *p* < 0.01, results not indicated were statistically non-significant.

**Table 7:** Correlations between DAS apathy and DASS depression among the different clinical groups.

		Depression
Clinical Group	Scale	Spearman’s Rho
**ADD**		
	DAS	
	Total Apathy	0.453 ***
	Executive Apathy	0.509 ***
	Emotional Apathy	0.220
	Initiation Apathy	0.276
**MCI**		
	DAS	
	Total Apathy	0.244
	Executive Apathy	0.346
	Emotional Apathy	0.099
	Initiation Apathy	0.151

Note 1: ADD (Alzheimer’s disease dementia), MCI (mild cognitive impairment), DAS (dimensional apathy scale), DASS (depression anxiety stress scale). Note 2: *** *p* < 0.01, results not indicated were statistically non-significant.

**Table 8:** Correlations between DAS apathy & DASS anxiety among the different clinical groups.

		Anxiety
Clinical Group	Scale	Spearman’s Rho
**ADD**		
	DAS	
	Total Apathy	0.296 **
	Executive Apathy	0.458 ***
	Emotional Apathy	0.061
	Initiation Apathy	0.063
**MCI**		
	DAS	
	Total Apathy	0.128
	Executive Apathy	0.296
	Emotional Apathy	0.016
	Initiation Apathy	0.006

Note 1: ADD (Alzheimer’s disease dementia), MCI (mild cognitive impairment), DAS (dimensional apathy scale), DASS (depression anxiety stress scale). Note 2: ** *p* ≤ 0.05, *** *p* < 0.01, results not indicated results were statistically non-significant.

**Table 9:** Correlations between DAS apathy & NPI apathy in ADD patients.

DAS	NPI Apathy
Total Apathy	0.476 ***
Executive Apathy	0.428 ***
Emotional Apathy	0.303
Initiation Apathy	0.595 ***

Note 1: NPI (Neuropsychiatric Inventory), DAS (dimensional apathy scale). Note 2: *** *p* < 0.01, results not indicated were statistically non-significant. Note 3: ADD patients: N = 41.

## Data Availability

For data availability and access, please contact: drmougias@gmail.com.
